# Implementation Interventions Used in Nursing Homes and Hospitals: A Descriptive, Comparative Study between Austria, Germany, and The Netherlands

**DOI:** 10.1155/2013/706054

**Published:** 2013-07-14

**Authors:** Helga E. Breimaier, Ruud J. G. Halfens, Doris Wilborn, Esther Meesterberends, Gunnar Haase Nielsen, Christa Lohrmann

**Affiliations:** ^1^Institute of Nursing Science, Medical University of Graz, Billrothgaße 6, 8010 Graz, Austria; ^2^Department of Health Services Research Focusing on Chronic Care and Ageing, Section of Nursing Science, Maastricht University, P.O. Box 616, 6200 MD Maastricht, The Netherlands; ^3^Department of Nursing and Management, Faculty of Business & Social Sciences, Hamburg University of Applied Sciences, Alexanderstraße 1, 20099 Hamburg, Germany; ^4^Department of Nursing and Health Sciences, Protestant University of Applied Sciences, Zweifalltorweg 12, 64293 Darmstadt, Germany

## Abstract

Translating guidelines into nursing practice remains a considerable challenge. Until now, little attention has been paid to which interventions are used in practice to implement guidelines on changing clinical nursing practice. This cross-sectional study determined the current ranges and rates of implementation-related interventions in Austria, Germany, and The Netherlands and explored possible differences between these countries. An online questionnaire based on the conceptual framework of implementation interventions (professional, organizational, financial, and regulatory) from the Cochrane Effective Practice and Organization of Care (EPOC) data collection checklist was used to gather data from nursing homes and hospitals. Provision of written materials is the most frequently used professional implementation intervention (85%), whereas changes in the patient record system rank foremost among organisational interventions (78%). Financial incentives for nurses are rarely used. More interventions were used in Austria and Germany than in The Netherlands (20.3/20.2/17.3). Professional interventions are used more frequently in Germany and financial interventions more frequently in The Netherlands. Implementation efforts focus mainly on professional and organisational interventions. Nurse managers and other responsible personnel should direct their focus to a broader array of implementation interventions using the four different categories of EPOC's conceptual framework.

## 1. Introduction

Nurses are expected to deliver care that is regularly updated with research findings, and the volume of new scientific evidence for good clinical practice is growing quickly. This research-based knowledge should be used, for example, as a basis for decision making [1] and for providing evidence-based care to promote positive patient outcomes [2, 3]. Legal regulations commit governments to providing their citizens with healthcare based on the acknowledged state of scientific knowledge; for example, in Austria, there is the Health Care Quality Act (Section 3) [4]. Regulations also require nurses to update their knowledge and skills according to the latest scientific findings in nursing science. In Austria, this is governed by the Nursing Act (Section 4, paragraphs 1 and 2) [5]. However, numerous studies over the past decade have highlighted a failure to routinely translate research findings into daily practice [6–11]. A large gap between what is known from research and what happens in practice still exists [10, 12], and introducing change is far from straightforward [13]. As a consequence of this theory-practice gap, patients may suffer unnecessarily from inconveniences such as pain, pressure ulcers, and/or prolonged hospitalization [11, 14]. 

Much effort has been undertaken to implement new knowledge to change nursing practice. Clinical practice guidelines are seen as one means by which new knowledge can be instilled in practice [15] in order to change the process and the outcome of care provided by allied medical professionals [16]. However, knowledge distillation and the publication of research findings or guidelines do not ensure that practitioners will use them in their daily clinical practice [17, 18]. Translating research into healthcare decision making and practice remains a considerable challenge [19]. There are several reasons why research results are not implemented successfully in clinical practice, including, for example, characteristics of innovations [20], insufficient education/information, or inadequate organisational support [21]. Over the past decade, a growing number of articles have discussed interventions that implement new knowledge in healthcare [22–26]. This is especially true for medical but also, to an increasing extent, for nursing care. These articles show, among other things, why the effectiveness of implementation interventions varies in healthcare [27–30]. Alanen et al. revealed, for example, that the method of implementing a hypertension guideline in Finnish health centres varied widely. Health centres categorized as implementers used multiple strategies to promote the adoption of the guideline. Health centres categorised as disseminators did little to facilitate their adoption [31]. Earlier research disclosed that methods utilized to implement guidelines in primary healthcare were usually directive and passive [32]. According to Kortteisto et al., profession also has an effect on the intention to use clinical guidelines in patient care, and the authors recommended the use of different strategies when clinical guidelines are targeted at different professional groups [33].

There is a broad array of implementation interventions from which nurse managers, for example, may choose to implement new knowledge in their respective practical fields. The Cochrane Effective Practice and Organisation of Care Review Group compiled a checklist with a short description of each intervention, which was then grouped into four non-mutually exclusive categories: professional interventions (e.g., distribution of educational materials), organisational interventions (e.g., changes in medical records systems), financial interventions (e.g., fee-for-service), and regulatory interventions (e.g., changes in medical liability) [34, 35]. These implementation interventions are thought to facilitate the use of known knowledge and help to overcome barriers to its adoption in clinical settings [34]. In a systematic review, Medves et al. focussed on dissemination and implementation strategies of practice guidelines for healthcare teams and team-based practice. They identified 88 relevant studies that investigated 16 dissemination and implementation strategies. Four of these strategies are ranked as organisational interventions, one as patient-oriented, and one as structural interventions. Ten of these strategies were aimed at healthcare professionals and included the distribution of educational materials and educational meetings as the most common strategies [36]. In an explorative study, Meesterberends et al. found that interventions used in disseminating pressure-ulcer guidelines in nursing homes of six European countries differed in terms of number (2–4) and type. The most common implementation interventions were the use of the Internet and the presentation of the guideline at national or regional seminars or congresses [37]. According to the typology of EPOC, both interventions belong to the category of professional interventions [35]. However, the evidence supporting the use of the electronic retrieval of healthcare information (including the internet) by healthcare providers to improve practice and patient care was found to be insufficient [38]. Furthermore, attendance at seminars or congresses is a passive educational approach which is considered a less effective implementation intervention [39]. 

This indicates that interventions used are not in line with existing evidence regarding implementation and may be one of the reasons why innovations are not implemented sustainably. To resolve this inconsistency, the effectiveness of implementation interventions aimed at instilling new knowledge into nursing practice must be understood. To this end, this study sets out to discover which implementation strategies are actually used in nursing practice and whether the most frequently used implementation strategies are indeed effective ones. This can help nurse managers to find the best methods for implementing new knowledge and/or guidelines. To date, however, only little is known about which interventions are actively used to implement guidelines in nursing—be it in hospitals or in nursing homes.

To shed more light on this issue, an international research group from Austria, Germany and The Netherlands set itself the objective of (1) determining the current range and rates of the interventions used for implementation purposes regarding guidelines in nursing in these countries and (2) exploring whether there are any differences in the interventions employed between the three countries. For this purpose, four care problems were chosen on an exemplary basis—discharge management (in hospitals), urinary incontinence (in nursing homes), and malnutrition and pressure ulcers (in both settings)—as these are among the more common care problems in hospitals and nursing homes [40–42].

## 2. Methods

### 2.1. Design

Using a cross-sectional design, data were gathered from nursing homes and hospitals in Austria (A), Germany (D), and The Netherlands (NL). 

### 2.2. Sample

Austrian, German, and Dutch hospitals and nursing homes were invited to participate in the study via email. In Austria, all nursing homes (692) and hospitals (268) nationwide were asked to participate. In Germany, for purposes of convenience, the survey was limited to the two federal states of Berlin and Hesse but included all nursing homes (Berlin: 228 and Hesse: 908) and hospitals (47 and 166, resp.). In The Netherlands, all 210 nursing homes and 70 hospitals which participated in the yearly National Prevalence Measurement of Care Problems (Landelijke Prevalentiemeting Zorgproblemen, LPZ) were invited to participate. The ethical principles of good scientific practice according to the Declaration of Helsinki [43] were observed throughout the entire research process. In a cover letter, participating institutions were informed about the study. Anonymity and confidentiality were assured, and voluntary participation was guaranteed. Contact information for further questions was made available for all three institutions carrying out this project. Approval for filling out the questionnaire was given by the participating institutions. Consent was assumed on the basis of a returned and completed questionnaire.

### 2.3. Procedure and Data Collection

The management and nursing directors of nursing homes and hospitals received an email notification about the intention to investigate interventions used to implement guidelines in nursing practice. The contact details of the institutions in Austria were acquired through an extensive internet search and from lists of nursing homes published by the Federal Ministry of Labour, Social Affairs and Consumer Protection. Contact details in Germany were acquired from lists published by the Ministry of Health, by the Public Inspecting Authorities for Nursing Homes of Hesse, and by searching the internet. In The Netherlands, all participants of the National Prevalence Measurement of Care Problems (LPZ) [41] were contacted. Two weeks later, all managers and nursing directors received detailed information about the purpose of the proposed study, the researchers' intention to disseminate the results, the data collection procedure, the return of the questionnaire, and the time requirements (45 minutes) for completing the questionnaire. A link to the online questionnaire was attached. The email contact and collection of the completed questionnaires were carried out by an external organisation (Flycatcher, Internet Research, Maastricht). Two reminders were sent to all managers and nursing directors, and the deadline was extended twice to increase the response rate. After completing the data collection, the anonymized final data file was forwarded to authors 1, 2, and 3 for analysis. Thus, anonymity was ensured for all participants. The managers and nursing directors were asked to forward the questionnaire to the nurse responsible for implementing guidelines in their institution and to return it by June 2008. 

#### 2.3.1. Instrument

 For comparison purposes, the modified typology of implementation interventions of the Cochrane Effective Practice and Organisation of Care (EPOC) Review Group [34, 35] was used for this study. The questionnaire included a total of four sets of questions based on the conceptual framework of four implementation interventions, namely, professional interventions, organisational interventions, financial interventions, and regulatory interventions [34, 35]. For each category of intervention, single interventions varying from bottom-up approaches (through participation) to top-down approaches (through instructions) were presented. Each intervention could be answered with *yes, no*, or* I do not know.* All questions were asked in relation to specific clinical domains. They were chosen on the basis of their prevalence in the three countries and the specific setting. In nursing homes, all questions relating to prevention of pressure ulcers, urinary incontinence, and malnutrition were asked. In hospitals, the clinical domain of *urinary incontinence *is less prevalent [44, 45] and was arbitrarily replaced by* discharge management*; its importance for hospitals has been pointed out previously [46–48]. The questionnaire consisted of 22 pages all together.

#### 2.3.2. Translation

The intervention part of the English EPOC-Checklist [35] was translated into German by authors 3, 5, and 6. Based on this translation, a German version of the instrument, addressing implementation issues of particular concern to nursing, was developed. This version was revised for Austria in order to take into account the localized nuances of Austrian German. In The Netherlands, the intervention part of the English EPOC-Checklist [35] was translated into Dutch by authors 2 and 4. The Dutch version was then translated into German by a professional translator. Both German versions were cross-checked, and unclear items were discussed within the research group. 

#### 2.3.3. Content Validation

The questionnaire was developed by the researchers, and a content validation procedure was performed in all three countries. Three experts in the field (nursing and medicine) from each country were asked to indicate whether the included items were relevant to implementation issues in hospitals and nursing homes. Possible answers ranged from agree completely, partly agree/partly disagree, to disagree completely. The instrument was amended using the feedback from the content validation procedure. In this course, the existence of nursing guidelines and expert standards was highlighted in the questionnaire, and some items concerning financial interventions were modified or removed completely.

#### 2.3.4. Pretest

After the process of content validation, an electronic web-based version of the questionnaire was developed. The questionnaire was piloted in three institutions per country to ensure that it was practical as well as easy to understand and read. The comments received were used to write the final electronic version of the questionnaire.

### 2.4. Analysis

Data were analyzed with SPSS 17.0. The percentage of each intervention was calculated by adding the answers of both the settings and the three care problems per intervention and dividing the result by the total number of institutions and then by three (care problems). The mean total number of each individual category of interventions and of the total interventions per setting and per care problem was calculated by adding the percentages of the respective individual items and dividing the result by 100. Next, the mean number of interventions of each category of intervention was calculated for each clinical domain and country. Differences between the three countries regarding the implementation interventions used were tested separately for each setting with one-way ANOVA. Due to the numerous tests, a *P* value of 0.01 was used for significance. The basis for the analysis was the net-response rates with *N*_total_ = 333.

## 3. Results

The response rates varied from 12.8% to 55.7%. In all countries, the response rate of hospitals was higher than nursing homes (between 8.3% and 28.6%). However, not all questionnaires were fully completed, so the net response of completed questionnaires (*n* = 333) was lower (8.7%–47.1%).

### 3.1. Implementation Interventions

In the three countries, various interventions were used during implementation, with a mean total of 19.6 interventions per setting per care problem. An overview of the interventions is shown in Table 1. The percentages presented are mean percentages calculated over the three care problems and over the institutions, which means that, for example, in Austria, professional internal written materials are used in 90% of the cases when implementing a guideline. The most frequently used implementation intervention is from the category of professional interventions, namely, providing written materials. On average this is used in 85% of cases. Other commonly used professional interventions (63%–66%) are internal consultations, internal audits and internal lectures. The organisational interventions used mostly (65%–78%) are changes in the patient record system, individual patient participation in treatment decisions, efforts to increase nurses' work satisfaction, regular use of assessment instruments, changes in structure and facilities of the institution, and management of patient complaints. 

*External electronic training* and *the use of telemedicine* are the professional and organisational interventions which are least used (≤12%). Financial interventions for nurses are rarely used (10%), while an average of 1.3 regulatory interventions is used per setting per care problem.

### 3.2. Implementation Interventions per Country

When looking at the mean total of interventions used per country, it becomes apparent that settings in Austria and Germany used slightly more interventions than those in The Netherlands (resp., 20.3, 20.2, and 17.3; *P* = 0.007) (Table 1). This is also true for the mean total of professional (A: 10.6, D: 10.3, NL: 8.2; *P* < 0.001) and organisational (A: 8.5, D: 8.3, NL: 7.0; *P* = 0.004) interventions. Regulatory interventions are, however, used less in Austria (A: 1.0, D: 1.4, NL: 1.5; *P* = 0.001).

The most frequently used professional interventions with significant differences between the countries are *internal audits, internal lectures, regular team meetings about a clinical topic, local opinion leaders*, and *written/manual reminders* (Table 1). *Internal written materials, internal consultations *and* external lectures* are used less in The Netherlands. The least frequently used professional intervention with a significant difference is *external conferences*. With regard to organisational interventions, the results show differences between the *regular use of assessment instruments, efforts to improve nurses' work satisfaction, case management, changes in right to access documentation*, and* changes in number of staff*. Compared to Austria and Germany, *financial incentives* for nurses are used more frequently in The Netherlands. Within the regulatory interventions, *inspecting authorities* and *insurance-related regulations* differ significantly (Table 1). 

The use of *internal written materials* ranks among the most frequently used professional interventions in all three countries, followed by *internal audits* and *internal lectures* in Germany, and *internal consultations*, *external conferences* as well as *internal audits* and *internal lectures* in Austria. In The Netherlands, the use of *internal written materials* is preceded by *regular team meetings about a clinical topic* and *manual reminders*. The three countries differ regarding the rank order of the most frequently used organisational interventions: in Austria these are *efforts to increase nurses' work satisfaction*, *changes in patient record systems* and *individual patient participation in treatment decisions*. In Germany the *regular use of assessment instruments* ranks first, whereas in The Netherlands *additional staff qualifications* is the third most frequent organisational intervention. The most often used regulatory intervention in Austria and The Netherlands is *inspecting authorities*, whereas in Germany *insurance-related regulations* is used most often (Table 1). An overview of the five most frequently used implementation interventions per country is provided in Table 2. In Austria and Germany, these are two professional and three organisational interventions. In The Netherlands, these are one regulatory, two professional, and two organisational interventions.

### 3.3. Implementation Interventions per Care Problem and Clinical Setting

These results are presented for each individual category of intervention.

#### 3.3.1. Professional Interventions

The number of professional interventions used in nursing homes and hospitals does not differ between the countries regarding the care problems of *pressure ulcers* and *malnutrition *(Table 3). However, there are significant differences regarding the care problems of *incontinence* in nursing homes and *discharge management* in hospitals. In both cases, the number of professional interventions is less in The Netherlands. Regarding *incontinence*, an average of 7.4 professional interventions was used to implement guidelines in Dutch nursing homes compared to 10.9 in Austria and 10.1 in Germany (*P* = 0.001). In terms of *discharge management*, an average of 2.1 professional interventions was used to implement guidelines in hospitals in The Netherlands compared to 9.6 in Austria and 9.3 in Germany (*P* < 0.001).

#### 3.3.2. Organisational Interventions

The evaluation of the organisational interventions yields a similar picture regarding differences between the settings and care problems in the three countries (Table 3). Regarding *incontinence*, an average of 6.3 organisational interventions were used to implement guidelines in Dutch nursing homes compared to 8.6 in Austria and 8.1 in Germany (*P* = 0.003). Regarding *discharge management*, an average of 3.2 organisational interventions was used to implement guidelines in hospitals in The Netherlands compared to 8.2 in Austria and 7.9 in Germany (*P* < 0.001).

#### 3.3.3. Financial Intervention

Financial incentives for the implementation of guidelines regarding care problems are available in only a few organizations (Table 3). In Dutch nursing homes they are used in total significantly more often (19.7%) than in Austrian (6.1%) and German (5.1%; *P* = 0.004) ones. This also holds true for the care problem of *pressure ulcers *(28.2%/6.5%/7.1%; *P* < 0.001). In hospitals, financial interventions are used less in Austria (6.7%) compared to Germany (17.0%) and The Netherlands (23.3%; *P* = 0.004) with the exception of *discharge management* (*P* = 0.041).

#### 3.3.4. Regulatory Interventions

The mean number of regulatory interventions is significantly lower in Austrian nursing homes (1.1) than in German (1.5) and Dutch ones (1.8; *P* = 0.002) (Table 3). The same is true for the individual care problem of *pressure ulcers* (1.2/1.5/1.9; *P* < 0.005) and *malnutrition* (1.0/1.6/1.8; *P* < 0.001). There is no significant difference between hospitals in the three countries, neither in total nor for the individual care problems. 

## 4. Discussion

Until now, “studies on the implementation of evidence based practice in nursing care make a very small contribution to the overall evidence on effective implementation” [49, p. 1163]. This study sought to gain an insight into ranges and rates of interventions used to implement nursing guidelines in Austria, Germany, and The Netherlands.

### 4.1. Ranges and Rates of Implementation Interventions

The results describing implementation interventions revealed that, on average, a broad array of implementation interventions was used. The most frequent ones were found within professional and organisational strategies. Financial and regulatory interventions were rarely used. This is in line with findings from the literature [37, 50] and, although indirectly, comparable with findings of a systematic review about the effectiveness of the dissemination and implementation interventions of practice guidelines for (multidisciplinary) healthcare teams. In this review, Medves et al. revealed that of the 16 interventions (classified according to the EPOC taxonomy), ten were professional and five were organisational/structural interventions [36]. Robertson and Jochelson conclude from their review that there is less research into organisational-level interventions than those targeted at individuals [51].

Among the five most frequently used interventions, the distribution of* written materials* as a professional intervention ranks first. This may be due to the advantage that printed educational materials can be distributed relatively cheaply and easily to a large number of professionals. Furthermore, its dissemination aims at improving the awareness, knowledge, attitudes, skills, and professional practice of nurses as well as patient outcomes [52]. This intervention is also among the five most frequently used interventions of a study investigating described implementation strategies in published abstracts [50], and it is the most commonly investigated one [36]. Although the majority of reviewed studies reported significant findings, it was not possible to attribute this intervention to the described effect. Grimshaw et al. concluded from their review that the distribution of written materials is a less effective method and that the addition of educational materials to other interventions did not seem to increase effectiveness [27]. This is supported by Farmer et al., who found a slightly beneficial effect on process outcomes but not on patient outcomes [52].

Four organisational interventions are ranked next: *changes in the patient record system*, *individual patient participation in treatment decisions*, *efforts to increase nurses' work satisfaction*, *and regular use of assessment instruments*. These measures do not force nurses to actively react to recommendations of an implemented guideline. Nonetheless, these measures seem to be a necessary basis which makes their use possible, in that, for example, it should be possible to record recommended care once it is carried out. Work satisfaction, which is associated with autonomy, good collaboration between nurses and physicians along with reduced job stress [53], was found to be an important individual characteristic of research utilization [54]. Out of these four most often used organisational interventions, *changes in the patient record system* was the only one included in Medves et al.'s work [36]. No conclusions could be drawn as to their effectiveness. In a former systematic review, however, Urquhart et al. found some limited evidence of effects on practice attributable to changes in nursing record systems [55]. A systematic review of reviews about organisational interventions found limited evidence in organisational interventions. None of the interventions produced consistent effects [56].

Additional frequently used interventions directed to professionals include *internal consultations, internal audits* and *internal lectures*. In these internally directed interventions, nursing managers may see a possible advantage in tailoring the specific content to the necessities of the respective setting in which they are delivered and/or a cost effective intervention possibility to introduce change. Often, audits are found to be investigated together with feedback as an implementation intervention. In general, their effect is consistently described as small to moderate for health practitioners [27, 28, 51, 57–60], yet not mandatory [61], and their effect could be increased where baseline adherence to recommended practice is low and when feedback is delivered more intensively [28]. An investigation by Dulko et al. in acute nursing care also showed their effectiveness [25].

*Internal lectures* are part of educational interventions which are usually regarded to be of limited [62] or no effect when used as a single intervention [39, 51, 57]. Simpson and Doig unexpectedly found that in-servicing (didactic lectures) was ranked highly, that is, as an effective practice change intervention, by participants [63]. In general, it can be said that educational meetings which include courses, conferences, lectures, workshops, seminars, and symposia can have a small effect on improving professional practice and healthcare outcomes [27, 39]; however, interactive educational strategies such as small-scale interactive meetings and workshops appear to be more successful in changing practice [51] and were consistently reported as effective [57, 64]. Yet, workshops, on average, were offered in less than half of the participating settings. Studies which reveal the effects of different educational implementation interventions showed only modest or moderate effects on processes or outcomes of care [27, 29, 39]. 

In general, financial and regulatory interventions were rarely used. Financial interventions, especially *financial incentives*, are seldom used to implement innovations in hospitals and nursing homes. The industrial sector, on the other hand, makes widespread use of financial incentives to encourage employees to achieve certain goals in their individual fields of responsibility. In the healthcare sector, however, this seems to be a rare measure to foster and implement quality improvement policies—at least in nursing. Actually, the use of financial incentives seems almost impossible considering that a separate budget for innovation implementation, which might be used for providing financial incentives, is very rare in the healthcare sector [16, 37]. Nevertheless, the effect of financial incentives was found to be inconclusive and provided no evidence that the magnitude of the incentive influenced compliance [57]. Flodgren et al. concluded that financial incentives may be effective in changing healthcare professional practice but not in improving patient outcomes [65]. Based on sparse observational studies found regarding regulatory interventions, Robertson and Jochelson summarized that regulation is associated with improvements in healthcare quality [51]. 

### 4.2. Comparison between the Three Countries

A count of the amount of implementation interventions shows that more interventions were used in Austria and Germany than in The Netherlands. However, when evaluating implementation interventions, limiting the perspective to mere numbers is not necessarily useful. Studies have revealed no relationship between the number of component interventions and the effects of multifaceted interventions on improvements in care [27]. It is still not clear how many and which combination of strategies are required to be successful [36].

For this reason, the authors of this study have focused their evaluation on the most common implementation interventions. In Austria and Germany, the five implementation interventions used most frequently are part of professional and organisational interventions with a ratio of 2 : 3. In The Netherlands they are part of professional, organizational, and regulatory interventions with a ratio of 3 : 1 : 1. The configuration of the used categories of implementation interventions shows a broader approach of implementation interventions in Dutch nursing homes and hospitals than in Germany and Austria. Moreover, in The Netherlands, interventions are also used which seem to focus on the ward-level, such *as regular team meetings about a clinical topic* and *written/manual reminders*, in addition to interventions applied on an institutional level. This might address nurses more directly and force them to react in a short time frame. According to Prior et al., the use of reminders consistently resulted in significant practical improvements, and computer-delivered reminders had a slightly greater effect than manual- or paper-based reminders [57]. Furthermore, in all but one of the five most used interventions per country, there was a significant difference between the countries. This exception is *individual patient participation in treatment decisions*, although ranked differently in the top-five list. Robertson and Jochelson found some evidence that providing educational materials to patients can help in the implementation of guidelines [51]. *Inspecting authorities* was among the five most used interventions in Dutch settings and is significantly more applied there than in Austrian and German settings. Inspection in its function of external oversight refers to surveillance and enforcement to ensure minimum standards are met [51]. The differences between the countries regarding these top-five interventions might be due to a higher awareness of the relevance of the process and steps of implementation in Dutch hospitals and nursing homes than in German and Austrian settings because of the longer history of nursing science in The Netherlands. Furthermore, The Netherlands started to develop national guidelines, for example, for pressure-ulcer prevention and treatment, as early as 1985 [66], whereas, in Germany, this process was started in 1999 with the development of *expert standards* (a type of guideline) [67], and education as the recommended implementation intervention [68]. In Austria, there exists no equivalent institution on a national level which is responsible for the development of national guidelines for relevant care problems in nursing. The difference regarding *inspecting authorities *might be due to the fact that, in The Netherlands, the Health Care Inspectorate regularly controls whether healthcare institutions meet their standards. Dutch healthcare settings are obliged to collect data about the so-called “norms of safe care”, for example, in the form of the annual prevalence survey, which was launched in 1998 [69]. 

### 4.3. Implementation Interventions per Care Problem

With regard to the amount of used professional and organisational interventions, no differences were found for *pressure ulcers* and *malnutrition *in the three countries. A possible explanation may be that these problems occur to a similar extent and that the amount of intervention reflects the magnitude of the care problems. This applies to malnutrition where prevalence rates are similarly high in nursing homes and hospitals: 25.7%/25.1% (A), 20.2%/22.8% (NL), and 23.0% in German nursing homes. In contrast, the prevalence rates of pressure ulcers are found to be highest in Dutch hospitals (10.2%) and lowest in German nursing homes (4.0%) [45]. In the field of incontinence care in nursing homes and discharge management in hospitals, there was a remarkably lower amount of professional and organisational implementation interventions in The Netherlands. It might be that these topics are regarded as less essential in Dutch nursing care. *Pressure ulcers*, *malnutrition,* and *incontinence* seem to be equally important areas in nursing care in Austria and Germany, and this might be due to their epidemiological relevance. Furthermore, since 2008 it has been mandatory for German nursing homes to implement what are known as *expert standards* [70], a kind of guideline focusing, among other things, on these care problems. Therefore, the high number of implementation interventions used in relation to those problems seems reasonable. Financial and regulatory interventions do not seem to play an important role regarding specific care problems, although they are used more frequently in The Netherlands relating to *pressure ulcers*.

Finally one should consider that the majority of the cited literature did not focus on the nursing profession but on healthcare professionals and medical doctors. Yet, interventions to implement guidelines should be tailored to different groups of stakeholders [15]. For this reason we should know the effectiveness of the used interventions.

### 4.4. Limitations

The results of this study might be limited due to low response rates—especially from nursing homes, which made comparison between hospitals and nursing homes difficult. Although low response rates are rather common nowadays [71, 72], several aspects could have had a negative influence, such as the use of a web-based questionnaire. Even though the questionnaire was developed in a very user-friendly way and tested for applicability, it could have been a problem for some respondents to fill in a web-based questionnaire. Also, the persons contacted might have considered the completion time for this task to be too long. Proposed participants were, however, notified beforehand of the forthcoming study. One further reason for low response rates may be that the questionnaire was distributed by a Dutch company, thus influencing possible participants from Austria and Germany. Furthermore, a high percentage of questionnaires was not fully completed; that is, the respondents stopped after the first few questions. In those cases, we decided to only use completely filled-out questionnaires.

Another limitation might lie in the fact that neither the characteristics of the person who filled out the questionnaire nor their role in the nursing team of each respective institution was identified. It is, therefore, possible that nurses in different positions with a variety of views about and responsibilities regarding implementation interventions filled in the questionnaire and this may, in turn, have influenced the results.

## 5. Conclusions

This study suggests that the most widely used implementation interventions to translate guidelines into nursing practice focus on professionals and organisations. There are only a few marked differences between the three countries, a fact which may be attributed to different familiarity with the field of nursing science and a different significance of problems in nursing care. After investigating the effectiveness of the most frequently used single interventions in the literature, it can be concluded that these are either of little or modest to moderate effect or that their effectiveness still lacks evidence. Furthermore, one should consider that the majority of the cited literature did not focus on the nursing profession. 

Nurses responsible for implementing nursing guidelines in practice should not focus mainly on a few implementation interventions with modest or even unknown effectiveness. On the contrary, at the beginning of an implementation process it might be worth considering what exactly has to be achieved and, based on this, selecting appropriate implementation strategies from all four different implementation categories. Yet to date, it is unknown how many implementation interventions, in which combination and from which EPOC-framework category [34, 35], would be effective in implementing a guideline into nursing practice. More research is necessary to explore whether a wide variety of implementation interventions is more effective than focussing on professional and organisational interventions alone.

## Figures and Tables

**Table 1:** Percentage of implementation interventions used per setting per care problem in Austria, Germany and the Netherlands.

	Total%	Austria%	Germany%	The Netherlands%	*F*-values*	*P *
(1) Professional interventions (22 items)						
Internal written materials	85	90	90	67	22.584	<.001
Internal consultations	66	71	71	47	13.433	<.001
Internal audits	65	62	80	43	22.050	<.001
Internal lectures	63	62	73	45	13.896	<.001
Regular team meetings about a clinical topic	58	51	55	74	7.026	.001
External lectures	56	63	64	31	19.055	<.001
Local opinion leaders	55	70	58	21	33.755	<.001
Written/manual reminders	52	54	42	68	8.206	<.001
Patient- and relative-mediated interventions	50	56	51	36	6.109	.002
Internal electronic materials	49	56	47	39	3.388	.035
External conferences	49	69	47	19	36.794	<.001
Internal workshops	46	44	46	48	.184	.832
External consultations	43	51	41	33	5.137	.006
External audits	42	34	52	39	5.562	.004
Computerised reminders	42	51	42	28	6.082	.003
External workshops	39	45	39	31	3.423	.034
Supervision	36	35	33	44	1.605	.202
Internal training sessions	35	30	37	39	2.045	.131
External training sessions	28	34	23	25	3.022	.050
Other internal interventions	16	13	17	21	1.262	.284
External electronic training	12	13	14	7	1.121	.327
Other external interventions	7	3	8	12	4.264	.015
Mean total number of professional interventions	*9.7 *	*10.6 *	*10.3 *	*8.2 *	*10.430 *	*<.001 *
(2) Organisational interventions (19 items)						
Changes in patient record systems	78	78	82	69	3.781	.024
Individual patient participation in treatment decisions	74	77	75	65	2.889	.057
Efforts to improve nurses' work satisfaction	70	82	78	34	41.201	<.001
Regular use of assessment instruments	67	54	84	58	30.744	<.001
Changes in structure and facilities of institution	66	70	68	56	3.806	.023
Management of patient complaints	65	66	70	56	2.314	.101
Additional staff qualifications	58	58	56	62	.458	.633
Changes in size and type of services of the institution	56	60	59	42	5.161	.006
Multidisciplinary teams to support nursing	52	53	47	57	1.354	.260
Changes in professional responsibilities	49	55	50	39	3.626	.028
Case management	41	40	34	57	6.754	.001
Changing the right to access documentation	38	48	38	19	9.656	<.001
Changes in number of staff	26	36	24	13	7.924	<.001
Changes in composition of staff	16	19	13	14	1.176	.310
Participation of patient groups	14	18	12	9	2.304	.101
Use of telemedicine	11	15	8	11	1.449	.236
Other patient interventions	10	9	10	12	.144	.866
Other structural changes	9	6	7	17	5.713	.004
Other personal changes	9	6	10	12	1.251	.288
Mean total number of organisational interventions	*8.1 *	*8.5 *	*8.3 *	*7.0 *	*5.595 *	*.004 *
(3) Financial intervention (1 item)						
Availability of financial incentives for nurses	10	7	8	21	8.070	<.001
(4) Regulatory interventions (4 items)						
Inspecting authorities	46	37	42	69	12.872	<.001
Insurance companies	34	25	45	31	7.473	.001
Law	33	28	40	27	3.093	.047
Other regulatory interventions	12	7	13	19	3.708	.026
Mean total number of regulatory interventions	*1.3 *	*1.0 *	*1.4 *	*1.5 *	*6.975 *	*.001 *

Mean total number of interventions used	**19.6**	**20.3**	**20.2**	**17.3**	**5.024**	**.007**

*df = 2, 330.

**Table 2:** Overview of the five most used implementation interventions per country.

Austria	Germany	The Netherlands
Internal written materials (professional intervention)	Internal written materials (professional intervention)	Regular team meetings about a clinical topic (professional intervention)
Efforts to increase nurses' work satisfaction (organisational interventions)	Regular use of assessment instruments (organisational interventions)	Changes in patient record systems (organisational interventions)
Changes in patient record systems (organisational interventions)	Changes in patient record systems (organisational interventions)	Inspecting authorities (regulatory interventions)
Individual patient participation in treatment decisions (organisational interventions)	Internal audits (professional intervention)	Written/manual reminders (professional intervention)
Internal consultations (professional intervention)	Efforts to increase nurses' work satisfaction (organisational interventions)	Internal written materials (professional intervention)

**Table 3:** Mean number of professional, organisational, financial and regulatory implementation interventions.

	Professional interventions (*n* = 22)		Organisational interventions (*n* = 19)		Financial intervention (*n* = 1)		Regulatory interventions (*n* = 4)	
	Nursing homes	Hospitals	Nursing homes	Hospitals	Nursing homes	Hospitals	Nursing homes	Hospitals
Pressure ulcers								
Austria	11.0	12.1	9.0	9.5	6.5%	8.0%	1.2	.8
Germany	10.9	13.2	8.8	9.7	7.1%	20.0%	1.5	1.3
The Netherlands	11.6	11.6	9.0	9.6	28.2%	39.4%	1.9	1.6
*F*-values*	.412	1.231	.122	.046	8.122	6.568	5.438	4.493
*P*-values	.663	.296	.885	.995	<.001	.002	.005	.013
Malnutrition								
Austria	10.3	9.1	8.3	7.5	6.5%	6.0%	1.0	.6
Germany	10.3	7.3	8.4	5.6	3.0%	11.4%	1.6	.7
The Netherlands	8.3	7.5	7.2	6.6	12.8%	27.3%	1.8	1.1
*F*-values*	3.876	1.533	2.022	1.887	2.401	4.108	9.820	2.819
*P*-values	.022	.220	.135	.156	.093	.019	<.001	.064
Incontinence								
Austria	10.9	—	8.6	—	6.5%	—	1.1	—
Germany	10.1	—	8.1	—	5.1%	—	1.5	—
The Netherlands	7.4	—	6.3	—	17.9%	—	1.6	—
*F*-values*	9.042		6.022		3.421		3.347	
*P*-values	<.001	—	.003	—	.035	—	.037	—
Discharge management								
Austria	—	9.6	—	8.2	—	6.0%	—	1.0
Germany	—	9.3	—	7.9	—	20.0%	—	1.0
The Netherlands	—	2.1	—	3.2	—	3.0%	—	.6
*F*-values*		33.352		16.124		3.582		1.957
*P*-values	—	<.001	—	<.001	—	.031	—	.146
Mean								
Austria	10.7	10.3	8.6	8.4	6.1%	6.7%	1.1	0.8
Germany	10.4	10.0	8.4	7.8	5.1%	17.1%	1.5	1.0
The Netherlands	9.1	7.1	7.5	6.4	19.7%	23.2%	1.8	1.1
*F*-values*	2.697	7.832	1.898	3.258	5.576	3.288	6.202	1.045
*P*-values	.070	.001	.152	.042	.004	0.041	.002	.355

*df (nursing homes) = 2, 212; df (hospitals) = 2, 115.
